# Perioperative Use of Vasoactive Agents in Patients Undergoing Gastrointestinal Surgery: A Protocol for a Scoping Review

**DOI:** 10.1111/aas.70326

**Published:** 2026-09-04

**Authors:** Pernille L. Jellestad, Morten Vester‐Andersen, Morten Hylander Møller, Mette Krag, Emilie S. Bækgaard

**Affiliations:** ^1^ Department of Anaesthesia and Intensive Care Rigshospitalet Hospital Copenhagen Denmark; ^2^ Herlev Anaesthesia Critical and Emergency Care Science Unit, Department of Anesthesiology Copenhagen University, Hospital Herlev‐Gentofte Copenhagen Denmark; ^3^ Department of Intensive Care Copenhagen University Hospital ‐ Rigshospitalet Copenhagen Denmark; ^4^ Department of Clinical Medicine, Faculty of Health Sciences University of Copenhagen Copenhagen Denmark; ^5^ Department of Anaesthesiology, Surgery and Trauma Centre Copenhagen University Hospital ‐ Rigshospitalet Copenhagen Denmark; ^6^ Department of Anaesthesia and Intensive Care Holbæk Hospital Holbæk Denmark

## Abstract

**Background:**

Perioperative hypotension in patients undergoing gastrointestinal surgery is a known and well‐described condition. Timely treatment of perioperative hypotension, regardless of the reason, is important in order to reduce the associated postoperative complications. The treatment consists of vasoactive agents and/or fluids; however, the ideal vasoactive agent in gastrointestinal surgery has yet to be identified.

**Methods:**

We will conduct a scoping review of studies assessing the use of vasoactive agents in patients undergoing gastrointestinal surgery. We will investigate in which gastrointestinal populations the use of vasoactive agents has been assessed, if the agents are used as part of a goal‐directed therapy protocol, evaluate the desirable or undesirable effects assessed, and if there are any differences between the agents assessed depending on the urgency of the surgery. The review will be developed in accordance with the Preferred Reporting Items for Systematic Reviews and Meta‐Analyses extension for Scoping Reviews (PRISMA‐ScR) statement.

**Results:**

Results will be presented descriptively.

**Conclusion:**

The outlined scoping review will provide a descriptive summary of the body of evidence on the use of vasoactive agents in patients undergoing gastrointestinal surgery.

## 1 Background

Perioperative hypotension is common and associated with postoperative complications such as myocardial or acute kidney injury, increasing morbidity and mortality [1, 2, 3, 4]. Timely treatment of hypotension is important regardless of the reason [5], and is often achieved through the use of vasoactive agents, fluids, or a combination. However, it is not known which strategy is most beneficial for patients undergoing gastrointestinal surgery [5].

A scoping review from 2024 evaluated the use of vasoactive drugs for perioperative hypotension in unselected surgical patients [6]. They found that studies concerning vasoactive agents for the treatment of perioperative hypotension varied considerably in all aspects. Populations were heterogeneous, interventions and exposures included multiple agents compared against themselves, each other, fluids or placebo, and studies reported primarily non‐patient‐centred outcomes, but did not report specifically on subsets of surgical specialties [6]. Previous research has highlighted negative effects of vasoactive treatment in patients undergoing gastrointestinal surgery, for example, anastomotic leakage, acute kidney injury and hyperlactatemia [7, 8, 9, 10] which we in this sub‐study aim to focus on investigating. The aim of the outlined scoping review is to provide an overview of the evidence on the vasoactive treatment of perioperative hypotension and associated outcomes in this patient population. We hypothesise that the body of evidence is limited.

## 2 Methods

This protocol has been prepared according to the Preferred Reporting Items for Systematic Review and Meta‐Analysis Protocol (PRISMA‐P) statement [11]. The outlined review will be prepared and reported according to the Preferred Reporting Items for Systematic Reviews and Meta‐Analyses extension for Scoping Reviews (PRISMA‐ScR) [11].

### 2.1 Research Questions

- In which gastrointestinal surgical populations has perioperative use of vasoactive agents been assessed, and how often are they used?
- Have vasoactive agents been assessed as individual interventions or as part of a goal directed fluid therapy algorithm?
- Have the agents been assessed as a bolus or an infusion regimen?
- Are there differences in the type of agents used according to the different gastrointestinal surgical populations, or according to the urgency of the surgery?
- Which desirable or undesirable outcomes have been evaluated?

### 2.2 Type of Studies

We will examine all study designs but will give priority to clinical randomised trials. Reviews, case reports, and letters will be excluded.

### 2.3 Type of Participants

We will include studies on both adults and children undergoing gastrointestinal surgery, who receive perioperative vasoactive treatment for hypotension. We will define gastrointestinal surgery as surgery related to the stomach, intestines, liver, biliary tree, pancreas/duct of pancreas and down the colon to the rectum. Studies in mixed surgical populations will be included if they include specific reports of gastrointestinal surgery. Studies on all other types of surgery, as well as studies on animals and healthy subjects, will be excluded.

### 2.4 Intervention and Comparator

We aim to include studies comparing any type of vasoactive agents with another agent, fluid treatment, no intervention or placebo, administered in the perioperative setting to treat hypotension during gastrointestinal surgery. Studies comparing two different fluid strategies (e.g., liberal vs. restrictive) are beyond the scope of this review and will be excluded, unless the strategies include one or more vasoactive agents.

### 2.5 Types of Outcome Measures

All reported outcomes, desirable and undesirable, will be described and classified according to patient‐centred or non‐patient‐centred outcomes. Authors will group these outcomes independently with inspiration by the Core Outcome Measures for Perioperative and Anaesthetic Care outcome set [12].

### 2.6 Electronic Searches

We will use a previously used broad search strategy where the included population was all non‐cardiac surgical patients [6]. We will update it to include all work published since the last updated search and manually restrict the population to gastrointestinal surgeries. In order to reduce publication and retrieval bias, we will not restrict our search by language, date or publication status.

We will systematically search all the Ovid MEDLINE databases, the Cochrane Library, Clinicaltrials.gov and the World Health Organisation (WHO) International Clinical Trials Registry Platform.

The final search string will be published with the manuscript. Before submitting the manuscript for review, we will conduct an updated search and include any relevant records. We will manually search systematic reviews and their reference lists for relevant studies (snowballing). Unpublished trials will be sought, identified and authors will be contacted for additional data, if relevant and possible.

### 2.7 Data Collection and Analysis

#### 2.7.1 Selection of Studies

At least two review authors will independently screen title and abstract of all studies identified in the search. The study selection process will be carried out using Covidence (https://www.covidence.org). All potentially relevant records will be assessed in full text for eligibility. Disagreements will be resolved through consensus or discussion with a third author. We will present a PRISMA flowchart of the study selection process [11].

#### 2.7.2 Data Extraction and Management

The study data will be extracted using a pre‐designed data extraction sheet. Information will include study characteristics (type of study, year of publication, duration and country), characteristics of participants (inclusion and exclusion criteria), the urgency and surgical site, type of intervention and comparator (if any) and outcomes. At least two review authors will independently extract information from the included studies.

#### 2.7.3 Strategy for Data Synthesis

Results will be presented descriptively and in tabulated form where appropriate. We will not provide a detailed assessment or critical appraisal of the individual studies.

## 3 Discussion

The outlined scoping review will provide an overview of the body of evidence on the use of vasoactive agents in the perioperative setting in patients undergoing gastrointestinal surgery. It will serve to highlight unanswered research questions and knowledge gaps in this field.

The strengths of the planned scoping review include the predefined protocol, the systematic search and compliance with PRISMA statements [11, 13]. Furthermore, we describe outcomes in relation to a pre‐defined and peer‐validated outcome set. Limitations include the risk of a high degree of clinical heterogeneity of the included studies regarding population, setting and intervention.

## 4 Conclusion

The scoping review proposed will provide an overview of current evidence available on the use of vasoactive agents to treat hypotension in a perioperative setting during gastrointestinal surgery. This may direct future research.

## Author Contributions

Conceptualisation: P.L.J., M.V.‐A., E.S.B. First draft: P.L.J. Review and editing: all authors.

## Funding

The authors have nothing to report.

## Conflicts of Interest

The authors declare no conflicts of interest.

## Data Availability

Data sharing not applicable to this article as no datasets were generated or analysed during the current study.
